# AI AND AGING: IDENTIFYING IMPORTANT LIVING ACTIVITIES FOR HEALTHY AGING IN SINGAPORE LONGITUDINAL AGING COHORT

**DOI:** 10.1093/geroni/igac059.2703

**Published:** 2022-12-20

**Authors:** Xin Zhong, Feng Yang, Wangyang Hu, Xinyi Gwee, Denise Chua, Qian Ling, Tze Pin Ng

**Affiliations:** A*STAR Singapore, Singapore, Singapore; Institute of High Performance Computing, Singapore, Singapore; National University of Singapore, Singapore, Singapore; National University of Singapore, Singapore, Singapore; A*STAR Singapore, Singapore, Singapore; National University of Singapore, Singapore, Singapore; National University of Singapore, Singapore, Singapore

## Abstract

Population aging in Singapore has encouraged the development of ambient smart communities to support healthy aging. Poor understanding of the living activities that address both clinical and biological concerns may plague efficient aging service designs and community health programs. We have designed a new computational workflow to identify important living activities for older adults in Singapore. We investigated innovatively three pillars of human life aspects: activities, clinical health, and biological health to identify living activities that are significantly associated with both clinical health and biological health. Cross-sectional data analyses were performed on 1356 community-living Chinese older adults of 65–80 years old in the Singapore Longitudinal Aging Study II (SLASII) cohort. 7 out of 29 living activities were found significantly associated with clinically healthy aging and showed improved prediction accuracy towards health status in machining learning schemes. Furthermore, biological age has been computed by screening and modeling 66 biomarkers. 15 out of the 29 living activities were found significantly associated with biologically healthy aging. Checking the overlapping living activities, we have found that physical exercise and cognitive-simulating activities are the most important activities for healthy aging: such as jogging regularly and reading, writing, and doing puzzles often. We regroup participants into active and non-active groups according to these two activities. The Keplan-Meier survival analysis showed statistically significant differences in survival time between the active and non-active groups (p < 0.001) in an 8-year longitudinal study. The workflow, results and biomarkers may provide references for future health program design improvement.

